# SUsPECT: a pipeline for variant effect prediction based on custom long-read transcriptomes for improved clinical variant annotation

**DOI:** 10.1186/s12864-023-09391-5

**Published:** 2023-06-06

**Authors:** Renee Salz, Nuno Saraiva-Agostinho, Emil Vorsteveld, Caspar I. van der Made, Simone Kersten, Merel Stemerdink, Jamie Allen, Pieter-Jan Volders, Sarah E. Hunt, Alexander Hoischen, Peter A.C. ’t Hoen

**Affiliations:** 1 Department of Medical BioSciences, Radboud University Medical Center, Nijmegen, 6525 GA the Netherlands; 2 European Molecular Biology Laboratory, European Bioinformatics Institute, Wellcome Genome Campus, Hinxton, Cambridge, CB10 1SD UK; 3 Department of Human Genetics, Radboud University Medical Center, Nijmegen, 6525 GA the Netherlands; 4 Department of Internal Medicine, Radboud Institute for Molecular Life Sciences, and Radboud Expertise Center for Immunodeficiency and Autoinflammation, Radboud University Medical Center for Infectious Diseases (RCI), Radboud University Medical Center, Nijmegen, the Netherlands; 5 Department of Otorhinolaryngology, Donders Institute for Brain, Cognition and Behaviour, Radboud University Medical Center, Nijmegen, 6525 GA The Netherlands; 6 Department of Biomolecular Medicine, Ghent University, Ghent, Belgium; 7 Laboratory of Molecular Diagnostics, Department of Clinical Biology, Jessa Hospital, Hasselt, 3500 Belgium

**Keywords:** Variant effect prediction, Rare diseases, Medical diagnostics, Computational pipeline, Immune response, Primary immunodeficiencies

## Abstract

Our incomplete knowledge of the human transcriptome impairs the detection of disease-causing variants, in particular if they affect transcripts only expressed under certain conditions. These transcripts are often lacking from reference transcript sets, such as Ensembl/GENCODE and RefSeq, and could be relevant for establishing genetic diagnoses. We present SUsPECT (Solving Unsolved Patient Exomes/gEnomes using Custom Transcriptomes), a pipeline based on the Ensembl Variant Effect Predictor (VEP) to predict variant impact on custom transcript sets, such as those generated by long-read RNA-sequencing, for downstream prioritization. Our pipeline predicts the functional consequence and likely deleteriousness scores for missense variants in the context of novel open reading frames predicted from any transcriptome. We demonstrate the utility of SUsPECT by uncovering potential mutational mechanisms of pathogenic variants in ClinVar that are not predicted to be pathogenic using the reference transcript annotation. In further support of SUsPECT’s utility, we identified an enrichment of immune-related variants predicted to have a more severe molecular consequence when annotating with a newly generated transcriptome from stimulated immune cells instead of the reference transcriptome. Our pipeline outputs crucial information for further prioritization of potentially disease-causing variants for any disease and will become increasingly useful as more long-read RNA sequencing datasets become available.

**Supplementary Information:**

The online version contains supplementary material available at 10.1186/s12864-023-09391-5.

## Background

The advent of next-generation sequencing (NGS) and the exponential increase in human genomes sequenced has caused a similarly strong increase in the number of genetic variants detected. The identification of novel genetic variants has outpaced the understanding of their functional impact. Since only a small fraction of all observed variants can be characterized clinically or by functional tests, there is a heavy reliance on computational methodology for prioritization. Several computational methods predict the effect of genetic variant effects on function such as PolyPhen-2 [1], SIFT [2], and MutPred2 [3]. Variant annotators such as the Ensembl Variant Effect Predictor (VEP) [4] and ANNOVAR [5] predict molecular consequences and integrate reference data and pathogenicity scores from different resources including dbNSFP [6].

Short-read RNA sequencing has provided us with the majority of knowledge we currently have about the transcriptome, but has some intrinsic limitations when it comes to discovery of alternative transcripts [7, 8]. Short read RNA sequencing is done on transcript fragments and the assembly into full-length transcripts is far from perfect, which has resulted in an incomplete reference transcriptome [9]. Long-read sequencing allows for the accurate elucidation of alternative transcripts [10] and long-read RNA sequencing datasets are proving that the human transcriptome has much more diversity than previously thought [11–13]. In addition, both short and long-read sequencing have shown that gene expression is highly variable in a context dependent manner, with divergent expression of transcripts expressed under different conditions (infection, stress, disease) or in different tissues or cell-types [14–17].

**Fig. 1:**
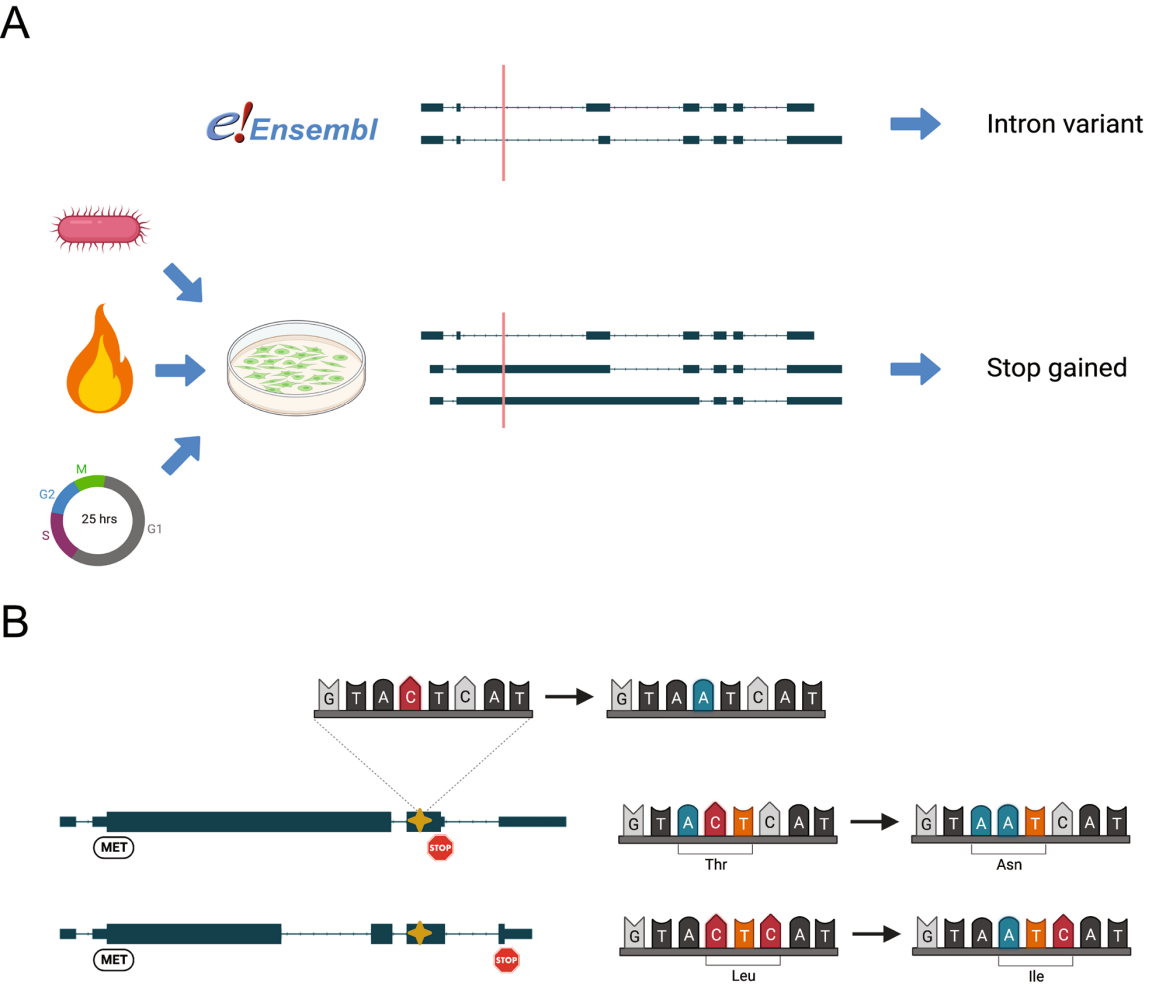
Premise for the creation of SUsPECT. (**A**) Some pathogenic variants may be missed without actual information about all alternative transcripts expressed in a relevant sample. A variant in a particular genomic position may be incorrectly predicted to be non-deleterious. (**B**) A variant at the same genomic position may cause a different missense variant in different transcript structures due to varying open reading frames per transcript

Some newly discovered transcripts result in open reading frames (ORFs) coding for novel proteoforms [18–20]. Knowledge on novel ORFs is key to predicting functional consequences of variants within them. There are several computational methods available to predict ORFs of these novel transcripts either based on sequence features [21–23] or homology to existing protein coding transcripts [24–26]. The prediction of ORFs on novel sequences is an essential first step for the detection of new proteoforms, as mainstream proteogenomics technologies for the discovery of proteoforms rely on databases with peptide sequences present in the predicted ORFs. Transcripts derived from long-read sequencing can provide better predictions of (novel) proteoforms (Fig. 1).

Current variant annotation tools do not take full advantage of the knowledge of novel transcripts because they work with precalculated pathogenicity scores calculated with respect to a fixed set of reference transcripts. This necessitates manual evaluation of the functional effects of variants on alternative proteoforms, since disruption of their function may have implications for clinical diagnosis and treatment. The pipeline presented here, SUsPECT (Solving Unsolved Patient Exomes/gEnomes using Custom Transcriptomes), is designed to leverage cell/tissue-specific alternative splicing patterns to reannotate variants and provide missense variant functional effect scores necessary for downstream variant prioritization. This pipeline was designed to be generalizable to any type of rare disease variant set paired with a relevant (long-read) transcriptome. For example, a researcher interested in annotating variants in a patient with a rare intellectual disability could consider using this tool along with a brain transcriptome dataset. We demonstrate the usefulness of this tool by reannotating ClinVar variants with a newly generated immune-related long-read RNA sequencing dataset.

## Results

### Analysis pipeline overview

**Fig. 2:**
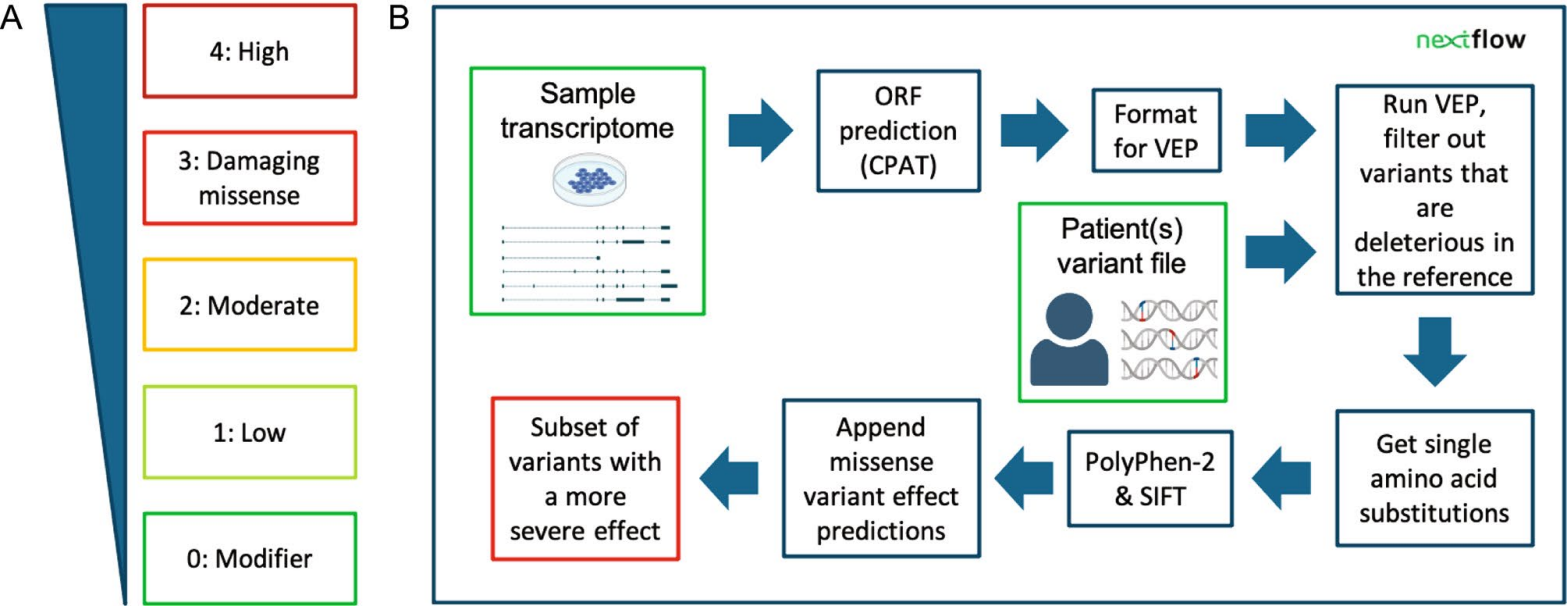
Reannotation with SUsPECT. (**A**) Defining “more severe”. The five categories of severity are modifier, low, moderate, damaging missense and high. We consider levels 3 and 4 to be deleterious, and thus potentially pathogenic. (**B**) The schematic of the pipeline

We developed SUsPECT to reannotate variants using custom transcriptomes (Fig. 2). This pipeline takes a custom transcriptome (GTF file) and a VCF file as input and returns a VCF file with alternative variant annotations for downstream evaluation and prioritization. SUsPECT predicts the ORFs in the alternative transcripts, calculates the molecular effects of the input variants with respect to these transcripts and predicts the pathogenicity of missense variants in the alternative proteoforms. SUsPECT displays subsets of variants predicted to have more severe effects when based on the custom transcriptome instead of the reference transcriptome. The predicted molecular consequences can be one of five severity levels, ranging from “modifier” to “high” (Fig. 2A). A schematic overview of the pipeline is presented in Fig. 2B. The main steps in the pipeline are:

- Validate pipeline input, including (1) an assembled (long-read) transcriptome in GTF format with novel transcripts. A long-read transcriptome assembly tool such as TALON will output a suitable file. (2) A VCF containing patient(s) variants.
- ORF prediction is performed on the transcripts that are not present in the human reference transcriptome.
- Ensembl VEP predicts molecular consequence annotations based on the user-provided set of transcripts/ORFs. Variants considered as missense in the user-provided transcriptome are reformatted and submitted to Polyphen-2 and SIFT.
- Polyphen-2 and SIFT calculate functional effect scores. These are reformatted and incorporated into the final VCF annotation file.
- A sub-list of variants that have a more severe molecular consequence in the custom transcriptome are provided in tabular format.

### A long-read sequencing transcriptome of stimulated peripheral blood mononuclear cells

We have generated long-read sequencing data on atypical, *i.e. in vitro* stimulated samples - provoking a strong expression response, to illustrate the use of the pipeline. We chose this dataset to exemplify less-studied tissues/conditions because novel transcripts are more numerous in these samples and SUsPECT is most likely to yield interesting results when the input transcriptome has many novel transcripts. Our custom transcriptome is based on long-read transcript sequences related to host-pathogen interactions and is derived from human peripheral blood mononuclear cells (PBMCs) exposed to four different classes of pathogens. We combined the transcript structures of all four immune stimuli and control samples for the reannotation. We identified a total of 80,297 unique transcripts, 37,434 of which were not present in the Ensembl/GENCODE or RefSeq reference transcriptomes. Relative abundances of novel transcripts were lower than of reference transcripts (Suppl. Figure 1). The custom transcriptomes resulted in prediction of 34,565 unique novel ORFs passing CPAT’s coding capacity threshold. The majority of transcripts had at least one ORF predicted (Suppl. Figure 2).

### Reannotation of ClinVar variants

Variants may be predicted to have a more severe molecular consequence in novel (non-reference) transcripts, but the functional and ultimately clinical implications remain unclear. To demonstrate that SUsPECT can suggest new candidate pathogenic variants associated with clinical outcomes, we reannotated ClinVar variants. ClinVar contains variants with clinical significance asserted by different sources. We hypothesized that ClinVar variants that were annotated as pathogenic and not predicted to be deleterious with the reference transcript annotation, but predicted deleterious with a (relevant) sample transcriptome, would support the utility of this pipeline.

We tested SUsPECT on a recent ClinVar [27] release (April 2022), excluding all variants that were annotated in ClinVar to be (probably) benign. We compared the predicted severity of the 776,866 variants using our custom transcript annotation versus the reference. After applying filters as described in the Methods section, 1,867 candidate variants remained. Of these variants, 145 were associated with monogenic immune-related disorders (Suppl. Table 1), which is significantly more than expected by chance (odds ratio = 5.46, p = 1.51 × 10^− 55^, Fisher’s exact test). This could indicate that annotation with an immune-relevant transcriptome is better suited for the identification of variants with an impact on immune function than annotating with a reference transcriptome. The strongest argument for the utility of this pipeline can be made with variants that are curated in ClinVar to be pathogenic rather than those of uncertain significance. After excluding variants of unknown significance (VUS) from the full candidates list, there are 90 variants remaining, of which 5 immune-related. These 90 variants had an enrichment of severity level 4 events (Suppl. Figure 3). An overview of the number of variants remaining after the different filter steps is given in Suppl. Figure 4.

**Fig. 3:**
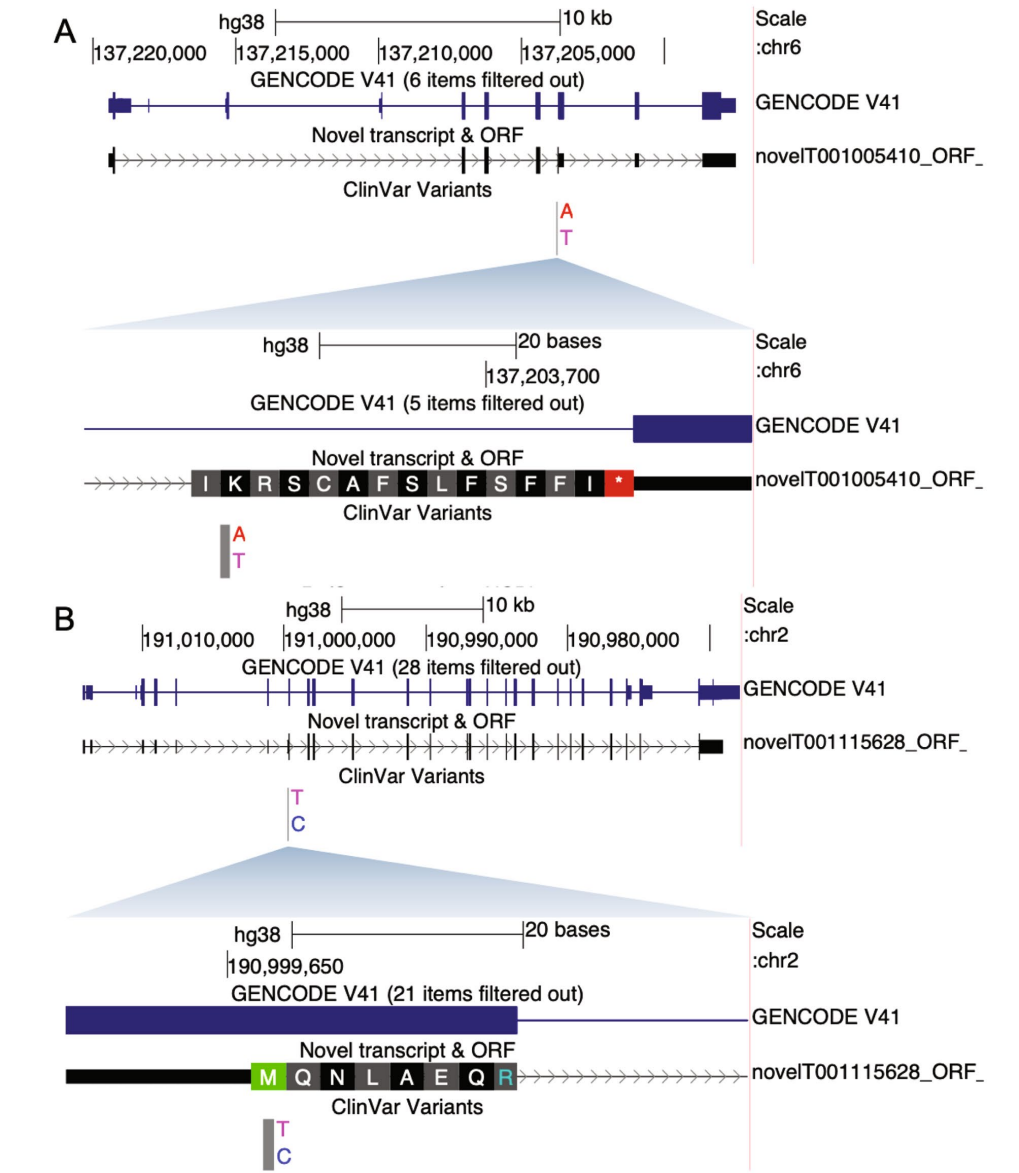
Two examples of ClinVar pathogenic variants being reannotated. Both variants were considered low severity variants when using hg38 reference transcriptome to annotate. (**A**) IFNGR1 whole view and close-up of region around the variant. Variant causes a stop-gain effect (K>*) in the custom transcript novelT001005410. (**B**) STAT1 whole view and close-up of region around variant. Variant causes a start loss (M > T) in the custom transcript novelT001115628

Five immune-related variants curated in ClinVar to be pathogenic were reannotated from a low severity molecular consequence in the Ensembl/GENCODE and Refseq transcript set to a moderate or high severity in our transcriptome (Table 1). Two were missense variants in the custom annotation and three were start-loss/stop-gain. We visualized the variants in the context of the transcript structures/ORFs on the UCSC genome browser. Two examples can be seen in Fig. 3. The variant in *IFNGR1* (dbSNP identifier rs1236009877) is associated with IFNGR1 deficiency. It is curated by a single submitter in ClinVar as ‘likely pathogenic’ using clinical testing. Annotation of the variant with reference transcripts results in a low severity (intronic variant) result, but results in a stop-gain variant (high severity) when annotating with our transcriptome. Our custom transcriptome contained multiple novel transcripts with a retained intron at the site of the variant, but only 1 of these transcripts had a predicted ORF in this intron. The particular transcript affected by this stop gained variant was found in all samples sequenced with minimum 3 and up to 10 supporting reads, indicating that it is unlikely an artifact. The predicted ORF extended 30 base pairs into the retained intron in the region of this variant. It was the most probable ORF for that transcript with a coding probability by CPAT of 0.934.

**Table 1:** Five ClinVar pathogenic immune-related variants annotated as low severity in the reference transcript set but high severity in the custom transcriptome

Variant	Location GRCh38	Allele	Gene	Consequence reference	Consequence custom	ClinVar condition	ClinVar evidence
rs80358236	1:172665641	C	*FASLG*	In-frame deletion	Start lost & in-frame deletion	Autoimmune lymphoproliferative syndrome	No assertion criteria provided. Citation; PMID: 8787672. No functional evidence.
rs1573262398	2:97724319	T	*ZAP70*	Benign missense	Missense (unknown)	Combined T and B cell immunodeficiency	Criteria provided, single submitter. No functional evidence, no citation
rs113994173	2:97733464	A	*ZAP70*	Intron	Missense (unknown)	Combined immunodeficiency due to ZAP70 deficiency	No assertion criteria provided. Citation; PMID: 20301777. No functional evidence.
rs387906763	2:190999647	G	*STAT1*	Benign missense	Start lost	Immunodeficiency 31 C	Criteria provided, single submitter. Citation; PMID: 21727188. No functional evidence.
rs1236009877	6:137203727	A	*IFNGR1*	Intron	Stop gained	Immunodeficiency 27 A	Criteria provided, single submitter. No functional evidence, no citation.

In addition, the variant in *STAT1* (dbSNP identifier rs387906763) was pathogenic according to the LitVar [28] literature mining tool and a clinical testing submission. It is a missense variant (Tgc/Cgc) in the reference annotation that is predicted by PolyPhen-2 to be benign. However, in one novel transcript it causes an M/T substitution, leading to loss of translation start site. Further inspection revealed that the transcript affected by the start-loss was expressed in *C. albicans*, *S. aureus* and PolyIC stimulated conditions by up to 6 supporting reads, but not in the control condition. STAT1 is previously described to be involved in the immune disease (chronic mucocutaneous candidiasis) linked to this variant by weakened response to *C. albicans* [29], which is a condition where this novel transcript was expressed. The ORF affected was the most probable ORF for that transcript and had a coding probability of almost 1 by CPAT.

## Discussion

SUsPECT predicts the functional consequences of genetic variants in the context of novel open reading frames predicted from a user-defined transcriptome. It is important to underline that the pipeline does not return a statement on the pathogenicity of variants. The pipeline simply brings new candidates forward for further interpretation; the user may choose to cross-reference the clinical phenotypes of the patients with the functions of the genes that the patients’ variants are found to disrupt. In our use case, ClinVar variants were used as they already have widely accepted annotations. However, 40% of ClinVar variants are of unknown significance, some of which are suspected to have some impact on clinical phenotype. Nearly 2% of these variants changed rating to be predicted as deleterious in our reannotation. As more people generate sample-specific transcriptomes to annotate variant sets, an increasing number of VUS may be classified as benign or deleterious.

Alternative splicing is known to increase the proteomic diversity, but it is less well understood how the novel transcripts contribute to the diversity of proteoforms and their function, and how these are impacted by genetic variants [30–33]. One of the most commonly used variant annotators, Ensembl VEP, predicts molecular consequences for variants in custom transcripts in standard formats, but lacks functional effect predictions for missense variants in those transcripts. Considering the well-established importance of missense variants on a variety of diseases [34–36], this presents a hurdle in the reannotation of variants with a custom transcriptome data.

We observed that many missense variants were predicted to have more severe effects when annotated based on custom transcriptomes. This may be due to the numerous new ORFs. Multiple ORFs passing CPAT’s ‘human threshold’ were often predicted per novel sequence; for our 37,434 novel transcript sequences we predicted 34,565 novel ORFs. Some proteogenomics tools choose the ‘best’ ORF per sequence, but we have decided to keep all that passed the probability threshold. We do not filter out non-coding genes when predicting ORFs, because some of them may still have protein coding capacity. Missense results implicitly depend on the confidence of the ORF predictions that are produced by CPAT. New deleterious missense variants will not be relevant if the predicted protein is not produced in the cell. Coding ability of novel transcripts is an area of active research [37–39] and new techniques to identify credible ORFs may be added to the pipeline as they become available. In the meantime, it may be prudent to validate interesting candidates using targeted proteomics techniques before establishing a genetic diagnosis.

SUsPECT is flexible; it takes transcriptomes from either short-read or long-read sequencing, PacBio or Oxford Nanopore, cDNA or direct RNA, as long as novel transcripts exist in the dataset. SUsPECT may produce the most comprehensive results if the transcriptome dataset comes from patient cells or tissues that are affected by the condition under study. However, it is also possible to use existing or newly generated long-read transcriptomes from relevant cells or tissues of healthy individuals, like we have demonstrated in the current work. The modularity of the tool means its components are also adaptable. The module that reads input can be updated as new (long-read) transcript analysis tools become available, which is useful considering new tools are actively being developed [40]. Its modularity facilitates incorporation of other functional effect prediction tools [41–44] than the currently implemented PolyPhen-2 and SIFT software. The current implementation and future extensions of SUsPECT may thus contribute to increase the diagnostic yield for disorders that are associated with transcripts expressed in specific tissues or under specific conditions.

## Conclusions

The full complexity of the human transcriptome is not represented in the current reference annotation. Analysing variants using alternative transcripts may aid in explaining missed genetic diagnoses, especially when disease or tissue-specific transcripts are used. SUsPECT puts genetic variants in the context of alternative transcript expression and can contribute to an increase in diagnostic yield. We used missense variants with ClinVar assertions of pathogenicity to demonstrate the potential of this methodology and have demonstrated a higher yield of missense variants are predicted to be deleterious. The enrichment of immune-related variants after reannotation suggests there is biological significance to these findings. Thus, long-read transcriptome data relevant to the disease of interest may not only improve our understanding of the ever-growing number of genetic variants that are identified in human disease context, but also aid in diagnoses for rare and/or unsolved disease [45, 46].

## Methods

### Severity classification

SUsPECT classifies variants according to their expected impact and their molecular consequence. Impact scores used by SUsPECT are based on the predicted molecular consequence groupings in Ensembl VEP (Fig. 2A) with higher numbers corresponding to more severe consequences: zero being equivalent to “modifier”, one to “low” severity, two to “moderate” severity, and four to “high” severity. SUsPECT uses Polyphen-2 predictions to distinguish between (likely) benign (score: 2) and (likely) deleterious (score: 3) missense variants.

### Additional filters for output variant list

SUSPeCT initial output is a list of variants with higher severity scores based on the custom transcriptome annotation compared to the reference annotation (homo_sapiens_merged cache version 104 which includes both Refseq and Ensembl/GENCODE transcripts). The variants that remain in the final list of “increasing severity” are filtered to retain only variants that are potentially interesting for establishing a disease diagnosis. Thus, the pipeline removes variants that are already considered deleterious based on the reference annotation, i.e. variants that already have scores of 3 or 4. An additional criterion was applied for missense variants. Missense variants for which the same amino acid substitution found in the custom and reference annotation are also removed. To reduce computational time further, missense variant alleles in novel sequences that are common (AF > 0.01) are removed. These filters are integrated in SUsPECT. For the use case described in this manuscript, missense variants present in the custom annotation that are predicted by PolyPhen-2 to be “benign” in both custom and reference annotation are removed. In our ClinVar example, we define “immune-related” variants as those variants that contain the string “immun” somewhere in the clinical description.

### Software details

A pipeline was built to streamline the process of variant prioritization using custom transcript annotation. The pipeline is written in Nextflow [47], using Ensembl VEP as the variant annotator. Each step of the pipeline runs Singularity/Docker containers pulled automatically from Docker Hub. The input of the pipeline is the sample-specific/non-reference long-read transcriptome in GTF format, variants in a VCF file, and a FASTA file of the genome sequence. It is designed for use with output from TALON [48].

First, the GTF file is converted to BED format with AGAT v0.9.0 [49]. ORFs for any novel sequences are predicted based on the BED annotation and FASTA genome reference using CPAT v3.0.4. CPAT output is converted to BED format with the biopj python package and filtered for a coding probability of at least 0.364, which is the cutoff for human ORFs recommended by the authors of CPAT [21]. Conversion from CPAT CDS to protein FASTA is performed with EMBOSS transeq v6.5.7. This ORF BED file is combined with the BED file of transcripts to make a complete BED12 file with ORF/transcript information. Then, we convert this BED12 file to GTF with UCSC’s bedToGenePred and genePredToGtf. The resulting GTF file is used for a preliminary annotation of the variants with Ensembl VEP to fetch variants predicted as missense in the custom transcript sequences. Next, variant filtering was performed as outlined in the previous section with the filter_vep utility distributed with Ensembl VEP as well as bedtools v2.30.0. The functional effect predictions from Polyphen-2 and SIFT are reformatted and one final run of Ensembl VEP (with the custom plugin enabled) integrates these predictions to the VCF. The output is the annotated VCF, as well as a VCF with the subset of variants predicted to have higher severity.

### Ex vivo PBMC experiments

Venous blood was drawn from a healthy control [50] and collected in 10mL EDTA tubes. Isolation of peripheral blood mononuclear cells (PBMCs) was conducted as described elsewhere [51]. In brief, PBMCs were obtained from blood by differential density centrifugation over Ficoll gradient (Cytiva, Ficoll-Paque Plus, Sigma-Aldrich) after 1:1 dilution in PBS. Cells were washed twice in saline and re-suspended in cell culture medium (Roswell Park Memorial Institute (RPMI) 1640, Gibco) supplemented with gentamicin, 50 mg/mL; L-glutamine, 2 mM; and pyruvate, 1 mM. Cells were counted using a particle counter (Beckmann Coulter, Woerden, The Netherlands) after which, the concentration was adjusted to 5 × 10^6^/mL. Ex vivo PBMC stimulations were performed with 5 × 10^5^ cells/well in round-bottom 96-well plates (Greiner Bio-One, Kremsmünster, Austria) for 24 h at 37 °C and 5% carbon dioxide. Cells were treated with lipopolysaccharide (*E. Coli* LPS, 10 ng/mL), *Staphylococcus aureus* (ATCC25923 heat-killed, 1 × 10^6^/mL), TLR3 ligand Poly I:C (10 µg/mL), *Candida albicans* yeast (UC820 heat-killed, 1 × 10^6^/mL), or left untreated in regular RPMI medium as normal control. After the incubation period of 24 h and centrifugation, supernatants were collected and stored in 350uL RNeasy Lysis Buffer (Qiagen, RNeasy Mini Kit, Cat nr. 74,104) at − 80 °C until further processing.

### RNA isolation and library preparation

RNA was isolated from the samples using the RNeasy RNA isolation kit (Qiagen) according to the protocol supplied by the manufacturer. The RNA integrity of the isolated RNA was examined using the TapeStation HS D1000 (Agilent), and was found to be ≥ 7.5 for all samples. Accurate determination of the RNA concentration was performed using the Qubit (ThermoFisher). Libraries were generated using the Iso-Seq-Express-Template-Preparation protocol according to the manufacturer’s recommendations (PacBio, Menlo Parc, CA, USA). We followed the recommendation for 2-2.5 kb libraries, using the 2.0 binding kit, on-plate loading concentrations of final IsoSeq libraries was 90pM (*C. albicans*, *S. aureus*, PolyIC, RPMI) and 100pM (LPS) respectively. We used a 30 h movie time for sequencing. The five samples were analyzed using the isoseq3 v3.4.0 pipeline. Each sample underwent the same analysis procedure. First CCS1 v6.3.0 was run with min accuracy set to 0.9. Isoseq lima v2.5.0 was run in isoseq mode as recommended. Isoseq refine was run with ‘--require-polya’. The output of isoseq refine was used as input for TranscriptClean v2.0.3. TranscriptClean was run with ‘--primaryOnly’ and ‘--canonOnly’ to only map unique reads and remove artifactual non-canonical junctions of each of the samples. The full TALON pipeline was then run with all five samples together using GRCh38 (https://www.encodeproject.org/files/GRCh38_no_alt_analysis_set_GCA_000001405.15/@@download/GRCh38_no_alt_analysis_set_GCA_000001405.15.fasta.gz). Assignment of reads to transcripts was only allowed with at least 95% coverage and accuracy. A minimum of 5 reads was required to keep alternative transcripts in the final transcript set (default of talon_filter_transcripts). GENCODE annotation (v39) was used by TALON to determine novelty of transcripts in the sample.

## Electronic supplementary material

Below is the link to the electronic supplementary material.

Supplementary Material 1

Supplementary Material 2

## Data Availability

SUsPECT is open source and freely available for download on GitHub (https://github.com/cmbi/SUsPECT). Raw PacBio sequencing data and transcriptome is available on EGA under accession number EGAS00001006779 https://ega-archive.org/search-results.php?query=EGAS00001006779.

## Data Availability and Requirements

-Project name: SUsPECT.

-Project home page: https://github.com/cmbi/SUsPECT.

-Operating system(s): Linux.

-Programming language: Python, DSL2.

-Other requirements: Nextflow, Singularity.

-License: Apache 2.0.

-Any restrictions to use by non-academics: no extra restrictions.
